# Comparative Study on Feeding Physiology, Metabolic Physiology, and Energy Budget of *Meretrix meretrix*, *Mactra veneriformis*, and *Ruditapes philippinarum* in the Tidal Flat Aquaculture Zone

**DOI:** 10.3390/ani15243543

**Published:** 2025-12-09

**Authors:** Longyu Liu, Yue Zhu, Chaozhong Xin, Jinmeng Bao, Fengbiao Wang, Shuai Han, Haopeng Hu, Xuan Zhang, Lei Li, Mei Jiang

**Affiliations:** 1 School of Marine Science and Environment Engineering, Dalian Ocean University, Dalian 116023, China; liulongyu1219@163.com (L.L.);; 2 East China Sea Fisheries Research Institute, Chinese Academy of Fishery Sciences, Shanghai 200090, China; alixyue@yeah.net (Y.Z.);; 3 Dalian Blue Carbon Engineering Technology Co., Ltd., Dalian 116000, China

**Keywords:** tidal flat aquaculture zone, *Meretrix meretrix*, *Mactra veneriformis*, *Ruditapes philippinarum*, energy budget, Rudong, China

## Abstract

(1) Exploring the potential impacts of *Mactra veneriformis* and *Ruditapes philippinarum* on the aquaculture of *Meretrix meretrix* in the tidal flat aquaculture zone is significant for the optimization of bivalve aquaculture structure. (2) In this study, we systematically compared the differences in feeding, metabolic physiological indicators, and energy budgets of the three bivalve species in the *M. meretrix* aquaculture zone of Rudong County, Jiangsu Province, under the feeding conditions of *Isochrysis zhanjiangensis* and *Platymonas helgolandica*. (3) The results indicated that feeding with *I. zhanjiangensis* could significantly improve the feeding efficiency of the three bivalve species, and *M. veneriformis* showed the strongest feeding ability. Under *I. zhanjiangensis* feeding conditions, the clearance rate [1.373 ± 0.177 L/(g·h)] and ingestion rate [9.803 ± 1.264 mg/(g·h)] of *M. veneriformis* were significantly higher than those of *M. meretrix* [clearance rate: 0.716 ± 0.046 L/(g·h); ingestion rate: 5.112 ± 0.328 mg/(g·h)] and *R. philippinarum* [clearance rate: 0.609 ± 0.101 L/(g·h); ingestion rate: 4.351 ± 0.725 mg/(g·h)] (*p* < 0.05). In terms of energy allocation, the scope for growth (SFG) of *M. veneriformis* [133.050 ± 15.705 J/(g·h)] was significantly higher than that of the other two bivalve species. *M. meretrix* [70.550 ± 2.133 J/(g·h)] had the highest energy utilization efficiency, while *R. philippinarum* [53.688 ± 7.548 J/(g·h)] had the lowest SFG due to high excretion losses. (4) In conclusion, *M. veneriformis* shows high production potential in the aquaculture system, and its potential impact on *M. meretrix* is greater than that of *R. philippinarum*, thus requiring close attention in aquaculture management.

## Simple Summary

In the tidal flat aquaculture zone, different types of bivalves are often grown together, but they compete for food and space. We wanted to understand how this competition affects the farming of the economically important bivalve, *Meretrix meretrix*. We studied the feeding, metabolism, and energy use of three common bivalves: *M. meretrix*, *Mactra veneriformis*, and *Ruditapes philippinarum*. We found that *M. veneriformis* has the strongest feeding ability and the highest potential for growth, meaning it could pose the biggest threat by consuming more food resources. In contrast, *M. meretrix* was the most efficient at using energy. We also discovered that feeding the bivalves with a high-quality microalga significantly improved their growth efficiency. This study helps farmers optimize their bivalve farming structure by suggesting which competing species to monitor closely and which feed to use, ultimately supporting more stable and productive bivalve harvests.

## 1. Introduction

China is the world’s largest aquaculture nation, with its marine aquaculture output accounting for over 50% of the global total [1]. Statistics indicate that in 2021, China’s total shellfish production reached 15.46 million tons, representing approximately 70% of its marine aquaculture output. From a structural perspective, filter-feeding bivalves such as oysters and clams constitute key marine aquaculture species in China [2]. Beyond their high economic value, filter-feeding bivalves influence nutrient cycling through physiological activities such as feeding, metabolism, and biodeposition. They provide vital ecosystem services, including water purification, mitigation of eutrophication, and enhancement of carbon sinks [3,4,5]. The physiological activities of filter-feeding bivalves, such as feeding and metabolism, serve as vital links connecting planktonic and benthic ecosystems. They also constitute fundamental parameters in physiological energetics and assessments of aquaculture capacity [3].

Tidal flats are coastal wetlands formed by sediments deposited in intertidal zones through tidal or river erosion [6]. Located at the junction of land and sea, these flats serve as vital habitats for the growth, reproduction, and survival of numerous macro-benthic organisms. Rudong County, Jiangsu Province, is located at the Yangtze River estuary, boasts an extensive coastline and abundant resources, making it a renowned *Meretrix meretrix* farming base in China [7]. *M. meretrix* belongs to the class Bivalvia, order Veneroida, and family Veneridae. *Mactra veneriformis* is a dominant species naturally distributed within farming bases, and *Ruditapes philippinarum* is an artificially introduced aquaculture species. The two bivalve species compete with *M. meretrix* for ecological niches and food resources, posing potential impacts on *M. meretrix* aquaculture.

In recent years, with the expansion of bivalve farming, the tidal flat environment has deteriorated. This has been accompanied by a series of ecological issues, such as high mortality rates, slow growth rates, and altered phytoplankton community structures, causing significant losses to the bivalve farming industry [8,9]. Currently, scholars have conducted extensive research on the physiological energy of species such as *R. philippinarum*, *Crassostrea gigas*, *Pteria penguin*, *Mercenaria mercenaria*, and *Crassostrea hongkongensis* [10,11,12,13,14]. These studies primarily focus on the physiological energy of single bivalve species or investigate the impact of specific environmental factors on bivalve energy budgets. However, no comparative studies have been reported on the energy budget of three bivalve species—*M. meretrix*, *M. veneriformis*, and *R. philippinarum*—which share similar ecological niches and often coexist in the same tidal flat aquaculture areas.

Therefore, this study measured and compared the feeding and metabolic physiology of these three species to evaluate their energy budget differences. We hypothesize that within the *M. meretrix* farming base, the presence of *M. veneriformis* and *R. philippinarum* compete with *M. meretrix* for food resources. Due to its gill structure and feeding mechanism, *M. veneriformis* is predicated to exhibit the highest feeding and growth potential, thus posing a greater competitive pressure on *M. meretrix* than *R. philippinarum*. The aim of this study is to assess the potential pressure from a physiological energetics perspective, providing a theoretical basis for optimizing the structure of the tidal flat bivalve farming and ensuring stable *M. meretrix* production.

## 2. Materials and Methods

### 2.1. Experimental Materials

**Figure 1:**
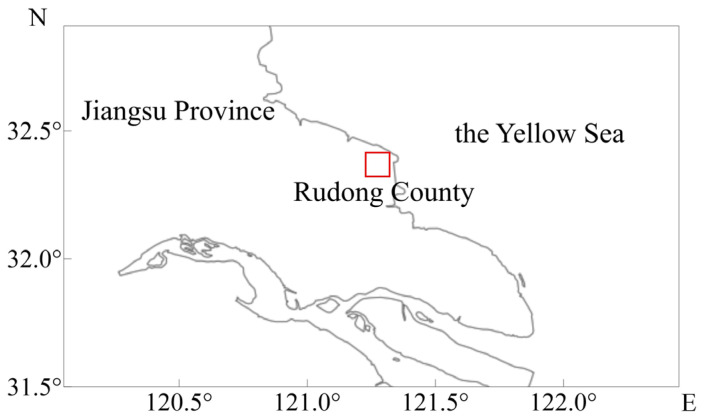
Map of sampling area.

The bivalves used in this experiment—*M. meretrix*, *M. veneriformis*, and *R. philippinarum* were collected from the *M. meretrix* farming base in Rudong County, Jiangsu Province (32°34′ N, 121°19′ E) (Figure 1). After collection, a total of 180 sexually mature, healthy, and similarly sized one-year-old bivalves (60 per species) were selected. Surface biofouling was removed, and the bivalves were temporarily retained in a flowing water experimental system at the East China Sea Fisheries Research Institute, Chinese Academy of Fishery Sciences, Shanghai, under a natural photoperiod of 12 h light: 12 h dark, for one week. During acclimation, the experimental seawater was filtered through a 0.22 μm membrane and UV-irradiated. Each species of bivalve was kept in a separate glass tank (60 × 50 × 50 cm), with three tanks in total. All tanks were equipped with automatic aerators to maintain dissolved oxygen levels above 6.5 mg/L. Temperature was maintained at 21.15 ± 0.82 °C. Salinity and pH were set to 20.45 ± 0.05 and 8.19 ± 0.21, respectively, based on water quality data from the *M. meretrix* farming base in Rudong County [7,15]. The bivalves were fed twice a day (at 09:00 a.m. and 5:00 p.m.) with pure cultures of *Isochrysis zhanjiangensis* and *Platymonas helgolandica,* and the feed amount was about 3% of the bivalves’ body weight every feeding time according to the conventional breeding method. Approximately 1/3 of seawater was exchanged daily, and the tanks were siphoned clean of feces and uneaten algae one hour after feeding. After acclimation, 5 individuals per replicate were selected for the experiment. The environmental conditions were the same as those during the acclimation period. Biological characteristics (Mean ± SD) for the three experimental bivalves are presented in Table 1. Shell length, height, and width were taken with an electronic digital caliper (0.01 mm precision; SIMCT, Shanghai, China); wet mass to 0.01 g (DIHENG, Shenzhen, China); shell dry mass and soft-tissue dry mass after 60 °C for 48 h.

*I. zhanjiangensis* and *P. helgolandica* are both commonly used in bivalve hatcheries and aquaculture in China. *I. zhanjiangensis* is a high-quality nutritive feed rich in polyunsaturated fatty acids [16], while *P. helgolandica* is a widely available species often present in natural phytoplankton assemblages [17]. They were purchased from Shanghai Guangyu Biological Technology Co., Ltd. (Shanghai, China) and cultured in F/2 Medium. Culture conditions were as follows: temperature 25.0 ± 1.0 °C, salinity 30.0 ± 1.0, light intensity 4000 lx, and a light–dark cycle ratio of 14L:10D. Seawater used for algal culture was sterilized by high-temperature treatment after filtration through a 0.22 μm pore size membrane.

### 2.2. Experimental Design

Two types of microalgae—*I. zhanjiangensis* and *P. helgolandica* were used to feed the following species: *M. meretrix*, *M. veneriformis,* and *R. philippinarum*. For each type of microalgae, three replicate groups and three blank control groups were established for each species of bivalve. For each parallel group, 5 individuals were placed in a thoroughly cleaned 2 L glass beaker containing 1.5 L of water. The two microalgal diets were standardized to an equal particulate organic matter (POM) concentration of 7.14 mg/L to ensure a valid comparison of dietary effects independent of food quantity. In total, 36 beakers (2 microalgae × 3 species × 3 replicates × 2 treatments [with bivalves vs. blanks]) were used. During the experiment, to prevent the sinking of microalgae, gentle aeration was maintained without affecting normal feeding of the experimental bivalves, ensuring uniform algal distribution in the water column and minimizing experimental error. The feeding experiment lasted for 2 h. After the experiment, place the bivalves in seawater filtered through a 0.45 µm membrane to collect feces within 24 h post-experiment, then determine particulate organic matter (POM) and total particulate matter (TPM). No pseudofeces were produced during the experiment. Use 2 L glass beakers to measure oxygen consumption rate and ammonia excretion rate, with 5 individuals placed in each beaker. Once experimental bivalves began filtering water normally, the beaker opening was sealed with plastic wrap to commence the experiment. Control groups without bivalves performed the same procedures. Throughout the experiment, a water bath was maintained to ensure consistent temperatures across all treatment groups. After 2 h, water samples were collected by siphoning for oxygen consumption and ammonia excretion rate measurements.

### 2.3. Experimental Methods

#### 2.3.1. Determination of Feeding Physiological Indicators

Ingestion rate [IR, mg/(g·h)] was defined as the mass of suspended particles consumed per hour per unit weight of the bivalve. It was expressed as the change in microalgae concentration in the water before and after the experiment, with the concentration of POM serving as the measurement indicator [18].

POM = W_65_ − W_450_ (1)

where W_65_ is the weight of GF/C glass fiber filters (Whatman, Maidstone, UK) used for filtering feed samples. The filters were burnt at 450 °C for 8 h in a muffle furnace beforehand. After drying in a 65 °C oven until constant weight, it was weighed using a balance with a precision of 0.01 mg (Sartorius BP211D, Göttingen, Germany). W_450_ is the weight of the W_65_ sample filter paper after incineration at 450 °C in a muffle furnace and subsequent cooling.

The IR calculation was conducted according to Griffiths [19]:

IR = (C_0_ − C_t_) × V/(W × t) (2)

where C_0_ and C_t_ represent the microalgae concentrations (mg/L) in the control and experimental groups at the end of the experiment, respectively; V is the experimental water volume (L); W indicates the dry weight of the soft tissue of experimental bivalves (g); and t is the duration of the experiment (h).

Clearance rate [CR, L/(g·h)] was defined as the volume of water filtered per hour per unit weight of the bivalve to remove suspended particles. It was computed by the formula according to Coughlan [20]:

CR = ln(C_0_/C_t_) × V/(W × t) (3)

where all parameters have the same meanings as above.

Assimilation efficiency (AE, %) was a parameter used to measure the absorption efficiency of the organic fractions contained in food (and excluding the inorganic fractions). It was computed by the formula [21]:

AE = (F− E)/[(1 − E) × F] × 100 E = W_1_/W_2_; W_1_ = W_65_ − W_450_; W_2_ = W_65_ − W_0_ (4)

where F is the percentage of ash-free dry weight in the feed, and E is the percentage of ash-free dry weight in the feces. W_0_ is the initial weight of the GF/C filter membrane, and the calculation method for F is consistent with that for E.

Fecal pellet production rate [FR, mg/(g·h)] was defined as the weight of feces excreted per hour per unit weight of the bivalve. The formula for calculating FR was as follows:

FR = IR × (1 − AE) (5)

where IR is the ingestion rate [mg/(g·h)], and AE is the assimilation efficiency (%).

#### 2.3.2. Measurement of Metabolic Physiological Indicators

The oxygen consumption rate [OR, mg/(g·h)] was defined as the amount of oxygen consumed per hour per unit weight of the bivalve. Dissolved oxygen (DO) was measured using an oxygen meter (model DO200A, YSI, Yellow Springs, OH, USA). The OR was calculated based on water changes in DO concentration before and after the experiment.

The OR calculation formula was as follows [22]:

OR = [(DO_0_ − DO_t_) × V]/(W × t) (6)

In the equation, DO_0_ and DO_t_ represent dissolved oxygen concentrations (mg/L) in the experimental water at the start and end of the experiment, respectively. V denotes the volume of experimental water (L). W represents the dry weight of the soft tissue of experimental bivalves (g), and t indicates the experimental duration (h).

The ammonia excretion rate [NR, mg/(g·h)] was defined as the amount of ammonia nitrogen discharged per hour per unit weight of the bivalve. Ammonia nitrogen (NH_4_^+^-N) was determined using the sodium hypobromite oxidation method [23]. The NR was calculated based on the changes in ammonia nitrogen concentration before and after the experiment.

NR was calculated by the formula [22]:

NR = [(N_t_ − N_0_) × V]/(W × t) (7)

where N_0_ and N_t_ represent the ammonia nitrogen concentrations (mg/L) in the experimental water at the start and end of the experiment, respectively; V is the experimental water volume (L); W is the dry weight of the soft tissue of experimental bivalves (g); and t is the duration of the experiment (h).

#### 2.3.3. Energy Calculation

The following energy conversion factors were used for energy budgeting in this study: 1 mg POM = 20.78 J; 1 mg O_2_ = 14.23 J; 1 mg NH_4_^+^-N = 24.83 J [24,25,26].

Energy balance equation for bivalves [27]:

C = R + U + P + F (8)

where C represents consumption energy [J/(g·h)], R denotes energy lost through respiration [J/(g·h)], U indicates energy lost through ammonium nitrogen excretion [J/(g·h)], P signifies growth energy [J/(g·h)], and F represents fecal excretion energy [J/(g·h)].

Scope for growth (SFG) refers to the total energy remaining for growth and reproduction after bivalves sustain basic life activities. The SFG formula is as follows [27]:

SFG = A − (R + U) (9)

where A is absorbed energy [J/(g·h)], defined as A = C − F.

### 2.4. Data Analysis

The experimental results are expressed as mean ± standard deviation (Mean ± SD). Data were processed using Excel 2024 (Microsoft Corp., Redmond, WA, USA) and analyzed statistically with SPSS 19.0 (IBM Corp., Armonk, NY, USA). One-way ANOVA and Duncan’s multiple range test were applied to the data, with *p* < 0.05 set as the significance level for differences. The Surfer 25 (Golden Software, Golden, CO, USA) was used to complete the map of sampling area and the results were plotted using Origin 2024 (OriginLab Corp., Northampton, MA, USA).

## 3. Results

### 3.1. Comparative Feeding Physiology of M. meretrix, M. veneriformis, and R. philippinarum

**Figure 2:**
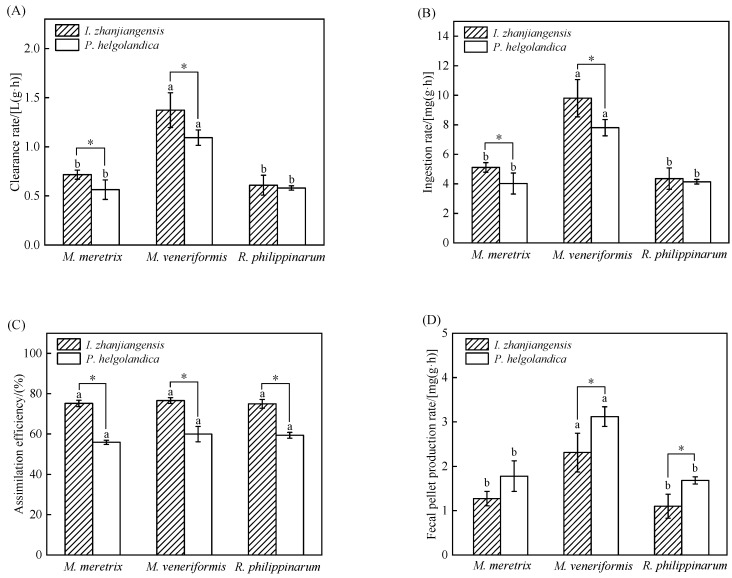
Feeding parameters (Mean ± SD) of *M. meretrix*, *M. veneriformis*, and *R. philippinarum*. (**A**) Clearance rate; (**B**) Ingestion rate; (**C**) Assimilation efficiency; (**D**) Fecal pellet production rate. Different letter superscripts indicate significant differences between the three bivalves fed with the same microalgae (*p* < 0.05), and “*” indicates significant differences between different microalgae fed to the same bivalve (*p* < 0.05).

The clearance rate (CR), ingestion rate (IR), assimilation efficiency (AE), and fecal pellet production rate (FR) of the three bivalves under the two dietary conditions are summarized in Figure 2.

When fed *I. zhanjiangensis*, *M. veneriformis* exhibited significantly higher CR and IR compared to *M. meretrix* and *R. philippinarum* (*p* < 0.05), while *R. philippinarum* showed the lowest values (Figure 2A,B). The AE of *M. veneriformis* was also higher than that of the other two species. Consequently, the FR of *M. veneriformis* was the highest among the three species (Figure 2D).

A similar pattern was observed when the bivalves were fed *P. helgolandica*. The CR and IR of *M. veneriformis* remained significantly superior to those of the other two species (*p* < 0.05), with *M. meretrix* showing the lowest CR (Figure 2A,B). *M. veneriformis* also maintained a higher AE (Figure 2C), resulting in the highest FR (Figure 2D).

Importantly, the species of microalgae significantly affected the feeding physiology of all three bivalves (*p* < 0.05). Both CR and IR were generally higher when bivalves were fed *I. zhanjiangensis* compared to *P. helgolandica* (Figure 2A,B).

### 3.2. Comparative Metabolic Physiology of M. meretrix, M. veneriformis, and R. philippinarum

**Figure 3:**
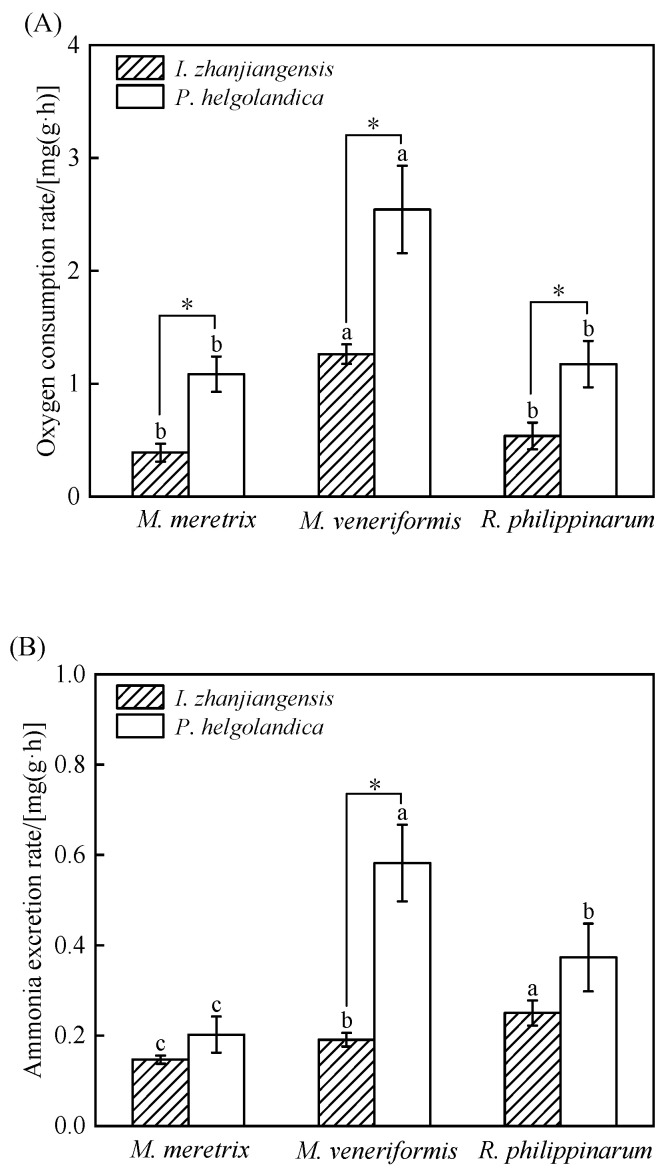
Metabolic parameters (Mean ± SD) of *M. meretrix*, *M. veneriformis*, and *R. philippinarum*. (**A**) Oxygen consumption rate; (**B**) Ammonia excretion rate. Different letter superscripts indicate significant differences between the three bivalves fed with the same microalgae (*p* < 0.05), and “*” indicates significant differences between different microalgae fed to the same bivalve (*p* < 0.05).

The oxygen consumption rate (OR) and ammonia excretion rate (NR) are presented in Figure 3.

Under *I. zhanjiangensis* feeding, the OR of *M. veneriformis* was significantly higher than that of *M. meretrix* and *R. philippinarum* (*p* < 0.05), while *M. meretrix* had the lowest OR (Figure 3A). In contrast, *R. philippinarum* exhibited the highest NR, followed by *M. veneriformis*, with *M. meretrix* showing the lowest NR (*p* < 0.05; Figure 3B).

When fed *P. helgolandica*, the OR of all three species increased markedly, but the interspecific pattern remained, with *M. veneriformis* displaying the highest value (Figure 3A). The NR in the *P. helgolandica* groups was also significantly higher than in the *I. zhanjiangensis* groups for all species, with *M. veneriformis* showing the highest NR and *M. meretrix* the lowest (*p* < 0.05; Figure 3B).

### 3.3. Comparative Energy Budget of M. meretrix, M. veneriformis, and R. philippinarum

The energy allocation strategies of *M. meretrix*, *M. veneriformis*, and *R. philippinarum* are presented in Table 2 and Table 3. Table 2 shows that, under *I. zhanjiangensis* feeding, respiratory and excretory losses accounted for a small proportion of the total ingested energy: 5.23% and 3.44% for *M. meretrix*, 8.81% and 2.32% for *M. veneriformis*, and 8.45% and 6.88% for *R. philippinarum*, respectively. The SFG of *M. veneriformis* was significantly higher than that of the other two species (*p* < 0.05), with *R. philippinarum* showing the lowest value.

Table 3 indicates that, when fed *P. helgolandica*, respiratory and excretory losses rose to 18.46% and 6.01% of total ingested energy for *M. meretrix*, 22.31% and 8.91% for *M. veneriformis*, and 19.40% and 10.77% for *R. philippinarum*. The SFG of *M. veneriformis* was also significantly higher than that of *M. meretrix* and *R. philippinarum* (*p* < 0.05).

## 4. Discussion

### 4.1. Comparative Feeding Physiology of M. meretrix, M. veneriformis, and R. philippinarum

CR and IR are crucial physiological indicators for studying filter-feeding bivalves, being highly susceptible to influences from both the organism’s condition and its surrounding environment. These factors include species type, size, temperature, salinity, water flow velocity, and suspended particulate matter concentration [28,29,30,31,32]. To investigate the influence of species type, this experiment employed a static water method in a controlled laboratory setting, effectively eliminating interference from factors like temperature, salinity, and flow velocity. The results showed that under feeding conditions with *I. zhanjiangensis* and *P. helgolandica*, CR and IR of *M. veneriformis* were significantly higher than those of *M. meretrix* and *R. philippinarum*, indicating stronger feeding physiological adaptability. This is consistent with findings by Teng Weiming et al. [33]. This difference may stem from the unique advantages of *M. veneriformis* in aspects such as gill structure, ciliary movement efficiency, or prey utilization mechanisms. The filter-feeding shellfish employ two primary feeding mechanisms: one relies on the coordinated movement of gill filaments and cilia to capture food particles, hydrodynamic transport of water with different food concentrations for bivalve filtration. While the other utilizes water flow to transport food particles through the gill filaments to the back of the gills and along the dorsal grooves into the labellum, selective feeding by bivalves [34,35]. *M. veneriformis* possesses inner and outer gill leaves on each side, with the inner gill leaf being larger and densely ciliated than the outer gill leaf. This structure enables it to capture more nutrients flowing into the mantle cavity [36], resulting in high CR and IR. Riisgård [37] obtained similar findings that *M. mercenaria* possesses large anterior lateral cilia capable of retaining microalgae larger than 4 µm with 100% efficiency, while bivalves with small or absent anterior lateral cilia exhibit retention efficiencies of only 75–85%.

The results of this experiment indicate that all three bivalve species exhibited higher IR in *I. zhanjiangensis* groups than in *P. helgolandica* groups. The cell diameter of *I. zhanjiangensis* is approximately 7 µm, while that of *P. helgolandica* is around 20 µm [38]. The optimal filter-feeding particle sizes for *M. meretrix* and *R. philippinarum* are 8 µm and 6 µm, respectively [39]. The particle size of *I. zhanjiangensis* falls within this range, which is one reason for the high IR of bivalves on *I. zhanjiangensis*.

### 4.2. Comparative Metabolic Physiology of M. meretrix, M. veneriformis, and R. philippinarum

Aerobic respiration is one of the fundamental metabolic processes in aquatic animals, and OR directly reflects their metabolic intensity. During respiration, aquatic animals consume dissolved oxygen in water to support metabolic processes and energy acquisition, thereby sustaining their vital functions. In this study, OR of *M. veneriformis* was significantly higher than that of *M. meretrix* and *R. philippinarum*. This difference is primarily determined by the biological characteristics of different bivalve species [40,41,42]. *M. veneriformis* exhibits a larger body size and typically inhabits deep mud-sand flat zones [41], whereas *M. meretrix* primarily resides in shallow marine mud-sand substrates [42], and the smaller-sized *R. philippinarum* can survive in diverse substrates [40]. Consequently, compared to *M. meretrix* and *R. philippinarum*, *M. veneriformis* requires a higher OR to provide sufficient energy for vital activities such as burrowing into the sand.

Ammonia is the final product of protein metabolism in marine invertebrates and is released mainly through respiratory organs [43]. NR reflects the level of protein metabolism in an organism [44] and is closely related to the quality and quantity of ingested food [45]. In this study, the NR of all three bivalve species in *P. helgolandica* groups exceeded those in *I. zhanjiangensis* groups, consistent with findings by Duong et al. [46]. This phenomenon may relate to nutritional differences between the two microalgae species. *P. helgolandica* (Chlorophyta) generally contains more crude protein than *I. zhanjiangensis* (Chrysophyta) [47,48]. When fed protein-rich Chlorophyta algae, bivalves may break down amino acids exceeding their immediate synthetic needs, leading to increased NR. Under both feed conditions, the NR of *M. meretrix* was significantly lower than that of *M. veneriformis* and *R. philippinarum*, suggesting either higher protein retention or more efficient amino-acid utilization. *R. philippinarum* exhibited higher NR and OR than *M. meretrix*, indicating a higher basal metabolic rate. From a physiological ecology perspective, such metabolic differences are often linked to species’ physiological structures and life history strategies [49]. These ultimately determine the SFG and affect the competitive performance of each species in aquaculture environments.

### 4.3. Effects of I. zhanjiangensis and P. helgolandica on Energy Budget of Bivalves

This study found that feeding *I. zhanjiangensis* and *P. helgolandica* produced significant and consistent effects on the energy budget of the same bivalve species (Table 2 and Table 3), with *I. zhanjiangensis* groups generally exhibiting higher energy utilization efficiency. *I. zhanjiangensis* belongs to the Chlorophyta phylum, lacking a cell wall and enclosed only by two layers of non-cellulosic plasma membranes. It is easily digested and absorbed, nutritionally rich, and abundant in various highly unsaturated fatty acids such as DHA and EPA, making it a recognized high-quality feed for bivalves [16]. In contrast, *P. helgolandica* from the Chlorophyta possesses relatively tough, fibrous cell walls [17,38] that are difficult for bivalve digestive enzymes to fully break down, resulting in generally lower AE. As described above, the diameter of *I. zhanjiangensis* falls within the optimal filter-feeding particle size range for all three bivalve species, significantly enhancing their CR and IR. Furthermore, AE of *I. zhanjiangensis* groups was markedly higher than that of *P. helgolandica* groups. The combination of high AE and particle size matching collectively resulted in the bivalves in *I. zhanjiangensis* groups exhibiting consistently higher feeding energy than those in *P. helgolandica* groups.

In this study, *I. zhanjiangensis* proved to be the better feed, giving higher energy utilization efficiency and growth potential. This provides strong physiological evidence for choosing effective feeds in the tidal flat bivalve aquaculture.

### 4.4. Comparative Energy Budget of M. meretrix, M. veneriformis, and R. philippinarum

SFG refers to the energy available for growth and reproduction after sustaining basic life functions in bivalves. It can be calculated as absorbed energy from ingested food minus respiratory and excretory losses. The results indicate that under two feeding conditions, the energy allocation strategies of *M. meretrix*, *M. veneriformis*, and *R. philippinarum* exhibit significant differences. These variations may be closely related to their species characteristics, physiological states, and habitat environments. *M. veneriformis* demonstrated the highest feeding energy and SFG under both feeding conditions, indicating its strongest growth and reproductive potential. This characteristic may be related to its highly efficient filter-feeding ability. For instance, its gill structure and feeding mechanism are better suited to environments with abundant food sources [33,36], significantly increasing energy intake. Additionally, its higher activity-related metabolic demands may also drive improvements in its energy utilization efficiency [41]. The high feeding capacity and growth potential of *M. veneriformis* can translate into competitive ability, potentially limiting the space and food resources of *M. meretrix*. In *I. zhanjiangensis* groups, *M. meretrix* exhibits the lowest ratio of respiratory and excretory energy, demonstrating a pronounced energy-conserving strategy. Its minimal energy losses may stem from its entry into a stable period of sexual maturity, where physiological metabolism stabilizes, and energy is prioritized for maintenance and reproductive reserves. Furthermore, its habitat in shallow muddy environments [42] may also contribute to reduced energy consumption. In contrast, while *R. philippinarum* has strong adaptability, it possesses the lowest SFG. Notably, their excretory energy accounts for a relatively high proportion of energy, suggesting potential physiological constraints in energy assimilation, such as high nitrogen metabolic load or low absorption efficiency [50]. This results in diminished net energy available for growth, potentially making its competitive pressure on *M. meretrix* relatively weaker than that of *M. veneriformis*.

Additionally, changes in environmental factors and physiological states significantly influence the energy allocation strategies of bivalves, manifesting as adjustments in feeding rates, alterations in metabolic rates, and slowed growth rates [51,52]. For instance, air exposure alters both metabolic and feeding rates in *P. penguin* [12], and elevated *p*CO_2_ significantly reduces the clearance rate and absorption efficiency while increasing the metabolic costs of *C. gigas* [52]. From an interspecific competition perspective, the potential threat of *M. veneriformis* to *M. meretrix* aquaculture is greater than that of *R. philippinarum*. Close monitoring of the population density of *M. veneriformis* is necessary in the tidal flat aquaculture zone to prevent its overpopulation. This study suggests that in practical aquaculture, the structure of bivalve farming should be reasonably arranged to optimize growth performance and reproductive output.

## 5. Conclusions

This study provides a comparative physiological foundation for understanding the potential interactions among three co-occurring bivalves in the tidal flat aquaculture. The key findings are threefold: First, the microalgal species significantly influenced bivalve performance, with *I. zhanjiangensis* proving more effective than *P. helgolandica* in enhancing bivalve growth efficiency. Second, interspecific differences in energy strategy were evident: *M. meretrix* demonstrated the highest energy utilization efficiency, whereas *M. veneriformis* exhibited the strongest feeding capacity and growth potential. Consequently, from a physiological energetics perspective, *M. veneriformis* poses a greater potential competitive threat to *M. meretrix* aquaculture than *R. philippinarum*. Accordingly, we recommend that the population density of *M. veneriformis* be closely monitored in the tidal flat aquaculture zone.

It is important to note that these conclusions are based on short-term physiological measurements. Actual competitive outcomes in the field are also influenced by factors such as population density, sediment characteristics, recruitment success, and seasonality. Therefore, field validation is recommended to confirm these physiological predictions and inform specific aquaculture management strategies.

## Figures and Tables

**Table 1:** Biological characteristics (Mean ± SD) of *M. meretrix*, *M. veneriformis,* and *R. philippinarum*.

Species	Number	Shell Length (mm)	Shell Height (mm)	Shell Width (mm)	Wet Weight (g)	Soft Tissue Dry Weight (g)	Shell Dry Weight (g)	Condition Index (%)
*M. meretrix*	30	41.17 ± 1.69	34.08 ± 1.64	21.18 ± 1.86	19.13 ± 2.06	0.808 ± 0.117	13.644 ± 2.063	5.949 ± 0.611
*M. veneriformis*	30	36.42 ± 1.34	31.94 ± 1.53	24.76 ± 1.26	15.95 ± 2.08	0.475 ± 0.038	5.828 ± 0.720	8.191 ± 0.615
*R. philippinarum*	30	36.25 ± 1.94	24.32 ± 1.20	16.14 ± 1.03	8.68 ± 1.27	0.365 ± 0.038	4.186 ± 0.501	8.808 ± 1.302

Condition index (%) = (Soft tissue dry weight)/(Shell dry weight) × 100%.

**Table 2:** Energy budget (Mean ± SD) of *M. meretrix*, *M. veneriformis*, and *R. philippinarum* in *I. zhanjiangensis* groups.

Species	C	R	U	F	SFG
*M. meretrix*	106.232 ± 6.825 ^b^	5.554 ± 1.143 ^b^	3.650 ± 0.212 ^c^	26.478 ± 3.371 ^b^	70.550 ± 2.133 ^b^
*M. veneriformis*	203.711 ± 26.261 ^a^	17.944 ± 1.240 ^a^	4.743 ± 0.376 ^b^	47.975 ± 9.032 ^a^	133.050 ± 15.705 ^a^
*R. philippinarum*	90.406 ± 15.056 ^b^	7.637 ± 1.665 ^b^	6.216 ± 0.702 ^a^	22.866 ± 5.622 ^b^	53.688 ± 7.548 ^b^

C, consumption energy [J/(g·h)]; R, respiration energy [J/(g·h)]; U, excretion energy [J/(g·h)]; P, growth energy [J/(g·h)]; F, feces energy [J/(g·h)]; SFG, scope for growth [J/(g·h)]. Different letter superscripts indicate significant differences between the three bivalves under the same parameter (*p* < 0.05).

**Table 3:** Energy budget (Mean ± SD) of *M. meretrix*, *M. veneriformis*, and *R. philippinarum* in *P. helgolandica* groups.

Species	C	R	U	F	SFG
*M. meretrix*	83.581 ± 14.693 ^b^	15.425 ± 2.220 ^b^	5.024 ± 0.995 ^c^	36.975 ± 7.167 ^b^	26.157 ± 5.478 ^b^
*M. veneriformis*	162.168 ± 11.528 ^a^	36.182 ± 5.514 ^a^	14.443 ± 2.116 ^a^	64.822 ± 4.616 ^a^	46.721 ± 6.939 ^a^
*R. philippinarum*	86.054 ± 3.225 ^b^	16.697 ± 2.932 ^b^	9.270 ± 1.863 ^b^	34.982 ± 1.691 ^b^	25.106 ± 2.719 ^b^

C, consumption energy [J/(g·h)]; R, respiration energy [J/(g·h)]; U, excretion energy [J/(g·h)]; P, growth energy [J/(g·h)]; F, feces energy [J/(g·h)]; SFG, scope for growth [J/(g·h)]. Different letter superscripts indicate significant differences between the three bivalves under the same parameter (*p* < 0.05).

## Data Availability

The data are included within the article.
